# Application programming in C# environment with recorded user software interactions and its application in autopilot of VMAT/IMRT treatment planning

**DOI:** 10.1120/jacmp.v17i6.6425

**Published:** 2016-11-08

**Authors:** Henry Wang, Lei Xing

**Affiliations:** ^1^ Department of Radiation Oncology School of Medicine Stanford University Stanford CA; ^2^ Department of Electrical Engineering Stanford University Stanford CA

**Keywords:** autopilot of treatment planning, VMAT, IMRT, SPORT, inverse planning

## Abstract

An autopilot scheme of volumetric‐modulated arc therapy (VMAT)/intensity‐modulated radiation therapy (IMRT) planning with the guidance of prior knowledge is established with recorded interactions between a planner and a commercial treatment planning system (TPS). Microsoft (MS) Visual Studio Coded UI is applied to record some common planner‐TPS interactions as subroutines. The TPS used in this study is a Windows‐based Eclipse system. The interactions of our application program with Eclipse TPS are realized through a series of subroutines obtained by prerecording the mouse clicks or keyboard strokes of a planner in operating the TPS. A strategy to autopilot Eclipse VMAT/IMRT plan selection process is developed as a specific example of the proposed “scripting” method. The autopiloted planning is navigated by a decision function constructed with a reference plan that has the same prescription and similar anatomy with the case at hand. The calculation proceeds by alternating between the Eclipse optimization and the outer‐loop optimization independent of the Eclipse. In the C# program, the dosimetric characteristics of a reference treatment plan are used to assess and modify the Eclipse planning parameters and to guide the search for a clinically sensible treatment plan. The approach is applied to plan a head and neck (HN) VMAT case and a prostate IMRT case. Our study demonstrated the feasibility of application programming method in C# environment with recorded interactions of planner‐TPS. The process mimics a planner's planning process and automatically provides clinically sensible treatment plans that would otherwise require a large amount of manual trial and error of a planner. The proposed technique enables us to harness a commercial TPS by application programming via the use of recorded human computer interactions and provides an effective tool to greatly facilitate the treatment planning process.

PACS number(s): 87.55.D‐, 87.55.kd, 87.55.de

## I. INTRODUCTION

A clinical treatment planning software is a closed system and a task in treatment planning is realized through a series of operations (mouse clicks and/or keystrokes) within the software platform. While automation of many clinical tasks is highly desirable — such as generation of a customized treatment plan report for documentation purpose or production of a clinically sensible volumetric‐modulated arc therapy (VMAT)/intensity‐modulated radiation therapy (IMRT) treatment plan — doing so is hindered by two major problems. First, there is a lack of means to “concatenate” the manual operations of the graphical user interface (GUI) of a commercial treatment planning system (TPS). Some vendors provide application program interface (API) toolkit, which is a set of routines, protocols, and tools for building software (e.g., in the form of a scripting), and this can be employed for certain applications.^1^, ^2^ But the availability and applicability of the API depend heavily on the vendor. On a more fundamental level, a programming environment that allows us to interact with the TPS, such as receiving data from the TPS, assessing the received data, and providing feedback to the TPS, is not generally available in API toolkit.

The purpose of this work is two‐fold. First, we investigate a technique to probe the GUI and data structure of a commercial TPS (or other clinical software system) in an independent C# programming environment. By doing so, we can interact with TPS continuously to extract the updated information of treatment planning. The need for interacting with the GUI of an application software or Web application is quite prevalent, and there are software tools developed for the Windows and UNIX/LINUX systems. Using these tools, for example, one can develop a test method to click a hyperlink in a Web application, type a value in a text box, or branch off and take different testing actions based on a value in a field. In computer science, automated tests that drive the application through its user interface (UI) to examine the functionality of UI controls are known as Coded UI Tests and are widely employed to verify that the whole application, including its user interface, is functioning correctly. Coded UI Tests are particularly useful where there is validation or other logic in the user interface, and are also frequently used to automate an existing manual test.

The second purpose of this work is to present an effective strategy to autopilot the VMAT/IMRT planning process by utilizing the functionalities of a commercial TPS and the capabilities provided by the Coded UI. By recording the mouse clicks/keyboard strokes as executable subroutines for specific tasks during planning, we program applications in C#, in which the recorded actions are called back to accomplish a designated task without the planner's intervention. Clinically, treatment planning is largely a trial‐and‐error process and relies heavily on user software interactions. While it is desirable to automate the planning process through the development of intelligent optimization algorithm,^(1,3–9)^ most systems are far from being ideal and multiple iterative interactions are necessary to obtain a clinically acceptable plan. There are multiple tradeoffs in plan selection as the inverse planning algorithm of a TPS generally contains a number of model parameters, such as the weighting factors for the involved structures. The influence of these parameters on the resultant dose distribution is not known until an optimization calculation is done, necessitating a manual trial‐and‐error determination of the final solution. Instead of attempting to improve the optimization algorithm, which is typically out of the control of a TPS user, the programming platform here allows us to develop a technique that is capable of mimicking a planner's interactive planning and decision‐making process to search for a sensible solution using a commercially available system. The utility of the approach in facilitating VMAT/IMRT plan optimization is demonstrated by using previously treated clinical cases.

## II. MATERIALS AND METHODS

### A. Recording a planner's action during planning as a subroutine for subsequent applications

Microsoft Visual Studio Coded UI is employed to record the operations of a TPS user.^10^, ^11^ Coded UI is targeted at providing UI accessibility and facilitating the automation of GUI manipulation. It provides a unique framework for application programming in C# or other programming environment (Coded UI property providers support both Win32 and NET programs), and allows us to probe, identify, and manipulate UI elements of another application such as TPS.

The application programming environment allows automation of the UI functionality testing and manipulations, and beyond this, definitions and implementations of functionalities that are independent of their respective implementations. Specific to medical physics applications, one of the important implications of the approach is that it enables us to access existing clinical software tools through a computer program and facilitate investigation of new ideas. This type of “plug‐in” software is also useful to streamline the execution of a series of tasks for improved workflow. The strategy may also assist otherwise distinct applications with sharing data, which can help to integrate and enhance the functionalities of the applications.

A commercial Eclipse TPS (Varian Medical Systems, Palo Alto, CA) is used in this study. We first construct a library that includes specifications for routines, data structures, object classes, and variables. Each of the commonly used human software interactions of mouse clicks and/or keyboard stroke (KS) is recorded using Visual Studio Coded UI automation engine and saved as action subroutine into the library. To be specific, in Table 1 we list some key examples useful for subsequent autopiloted planning.

**Table 1:** A list of some useful recording and playback actions for autopiloted planning

*Function*	*Eclipse Operation*	*Action in Coded UI & Application in C#*
3D dose calculation	F5	Record the “F5” and playback the code when dose distribution needs to be updated.
Open an optimization window	F10	Record the “F10” and playback the code to change the Eclipse optimization parameters.
Adjust weighting factors or other parameters	Manually entering values into the textbox after clicking “F10”	Record the action of moving the mouse indicator to the textbox and entering new values. Playback with new input values.
Start an optimization	Click “Optimize” button after clicking “F10”	Record the action of clicking “optimize”. Playback when Eclipse optimizer needs to be executed with updated optimization parameters.
Export DVH to a file	Pull down the textbox “show DVH view” and select “export DVH in tabular format”	Record the DVH export process. Call the subroutine when updated Eclipse DVHs of the involved structures are needed.
Create a Boolean structure of two structures	Go to “Contouring” section, identify the structures, and perform a Boolean operation	Record the process (including “and”, “or”, “xor”, “sub”, and “not”). Call the subroutine when a Boolean structure needs to be created.
Convert an isodose region into a structure	Right clicking “dose” on the left side menu and select “convert isodose level to structure”	Record the process. Playback to iteratively refine an Eclipse‐optimized plan.
Refresh patient information	Clicking “Save All” and “Reload All” under the file pull‐down menu	Record the action. Call the subroutine when existing patient information needs to be refreshed or to move the planning to next stage.

### B. Playing back stored action subroutines

A C# programming environment is employed to utilize the recorded human TPS interactions to facilitate VMAT/IMRT planning process. In this environment, execution of a planning action described in Table 1 is realized by simply calling the recorded subroutine(s). A planning task is accomplished by executing a chain of prerecorded modules, as well as syntax that analyze the intermediate results for algorithmic decision‐making. To illustrate the utility of the proposed programming technique, the following presents a specific implementation of an autopiloted VMAT/IMRT planning.

### C. Autopiloted VMAT/IMRT treatment planning

**Figure 1:**
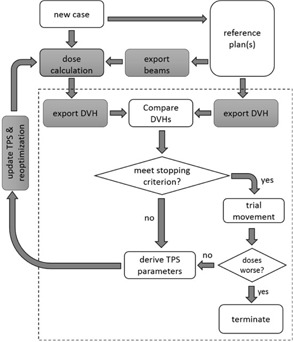
An architectural overview of the autopiloted VMAT/IMRT plan optimization scheme implemented using the proposed technique. An outer‐loop calculation (indicated by the dashed rectangular box) analyzes the generated Eclipse plan and feeds the Eclipse optimizer with updated parameters for improved dose distribution. The gray boxes are realized through the use of recorded Eclipse actions stored in a library.

Figure 1 shows the flowchart of an autopiloted VMAT/IMRT plan optimization process with the use of recorded Eclipse actions. After the planning target volume (PTV) and other involved structures are segmented, a reference plan with similar anatomy and prescription is chosen from a library to guide the search for a clinically sensible treatment plan. Here, some predefined geometric criteria are employed for the selection of reference plan. Similar to the work done by Chanyavanich et al.,^(12)^ the images of current case are overlaid with a candidate reference plan from a library of previously treated patients. The candidate reference plans are first filtered according to anatomical site, physical target volume(s), corresponding structure names, and prescribed dose. For each structure, the signed difference of the contour points of the current and reference plans is computed.^(13)^ In computing the signed difference, we first introduce a polar coordinate system with its origin located at the center of mass of the structure. Ray lines starting from the origin are introduced with an angular resolution of 2.5°. The points for a ray line to be in and out of the structure are recorded (for the PTV, generally, the ray intercepts with its contour once). The signed difference of an intercepting point is given by subtracting the radial distance of the point in the current case from that of the reference case. A plan is not considered as a good reference if the signed difference of any intercepting point in any structure is greater than 1–3 mm for a small structure such as the optic nerve and 5–15 mm for a large structure such as the skin contour. While the metric can certainly be further improved by introducing some heuristic weightings of the structures and points in the future, the scheme here captures the main features of the similarity assessment. In an ideal situation for two identical cases, the signed difference for all points becomes zero. In our study, a visual inspection of the coincidence of the current and reference cases is also performed to ensure the quality of selected reference case.

Generally speaking, the computational effort could be reduced if the information gained during the course of solving an optimization problem closely related to the current one is utilized in solving the problem at hand. In order to speed up the calculation, in this study, the beam and optimization parameters of the reference plan are used as the starting point of optimization, which is often referred to as a “warm‐start” of optimization. However, it is noted that a “warm‐start” is not necessary to find the optimal plan. Our calculation proceeds in analogous to the planning process of a human planner, with the resultant dose distribution assessed by a decision function each time after the Eclipse optimization is done.^(35)^ The algorithm is formally described by the following equations:

(1) $$\begin{array}{rl} & opt\;TPS\;objective\;function\;with\;constraints\\ & s.t.\;\left|d_{\sigma j}-d_{\sigma j}^{ref}\right|<\varepsilon_{\sigma }\;\;\;(\sigma \in 1,2,\ldots ,N;j\in 1,2,\ldots ,J) \end{array}$$

where the optimization of TPS objective function with consideration of constraints is done on the Eclipse TPS, $d_{\sigma j}$ and $d_{\sigma j}^{\text{ref}}$ are the doses of the j‐th segment of the DVH curve of structure σ for the current and reference plans, respectively. $\varepsilon_{\sigma }$ is the allowed deviation defined for the DVH segment of the structure σ.

The autopiloted plan scheme involves the following key steps: 1) optimizing the Eclipse plan, 2) comparing the Eclipse solution with reference data, 3) deriving a new set of Eclipse optimization parameters, and 4) updating the Eclipse optimization parameters and reoptimizing the beams. The DVHs of the involved structures are exported into a .csv file upon completion of an Eclipse optimization using a prerecorded Eclipse function (see Materials and Methods section A). The file is then read into the C# program and compared with the corresponding DVHs of the reference plan. The weighting factors of Eclipse, which represent the priorities of dosimetric objectives of different structures, are assessed based on the difference between current and reference DVHs. For computational purpose, the DVH file is discretized with the maximal dose resolution of 0.001 Gy. The adjustments of the weightings are made toward the direction of decreasing the discrepancy between the two DVHs. Note that, while $D_{\text{min}}$, $D_{\text{max}}$, and $D_{\text{mean}}$ are stored in the headers of the DVH file for each structure, dose volume constraints such as $V_{50}$ are listed in a tabular format following the headers. A subroutine is thus written to extract the dose and volume values from the DVH file for each structure. The autopilot planning process stops if the constraints in Eq. (1) with predefined $\left\{\varepsilon_{\sigma }\right\}$ are met (in our calculations, the value of $\varepsilon_{\sigma }$ is set to be 3% of the value in the reference plan) or if the number of iterations of the outer‐loop optimization is greater than 50. To ensure that the autopiloted planning does not stop at the reference plan when there is a room for improvement, at the end of calculation, we let each resultant DVH segment to make a trial movement toward better PTV coverage or organ at risk (OAR) sparing, even if the benchmarking goal of the segment set by the reference plan is met. The trial movement is accepted if the dose to any of other DVH segments is not worsened. By means of this last step, the autopilot process is made less dependent on the “perfect selection” of the reference plan.

The introduction of an outer‐loop decision function based on prior clinical experience forms the basis for the autopilot algorithm. The iterative interactions of the decision function and TPS pilots the search toward a clinically sensible solution. From the perspective of optimization, the strategy here is similar to a sequential optimization of an overall objective,^14^, ^15^ but the objective functions for the two stages (i.e., the TPS optimization and the outer‐loop determination of the TPS parameters) are different. This process is along the line of our earlier work in automated weighting factors and model parameters determination.^6^, ^16^, ^17^ Instead of simply using prior knowledge extracted from previous clinical treatment plan(s) as a “class solution” or as upper/lower bounds to examine the results of the optimization calculation,^18^, ^19^, ^20^, ^21^, ^22^, ^23^, ^24^ in our approach, the reference information is utilized throughout the plan selection process. During the process, the parameters used in the Eclipse optimizer are constantly updated through the comparison of current and prior knowledge characterized by reference plans. Generally speaking, it is a fundamental rule of estimation theory that the use of prior knowledge will lead to a more accurate estimation. An inclusion of even partial information could lead to more effective search of the solution space and eliminate some unnecessary uncertainties in the estimation process.^25^, ^26^, ^27^

### D. Case studies

**Figure 2:**
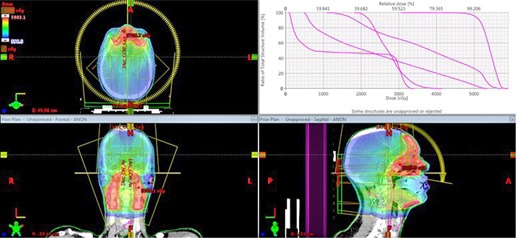
Isodose distributions in axial, coronal, and sagittal planes and DVHs of the involved structures of the reference for the first head and neck plan.

**Figure 3:**
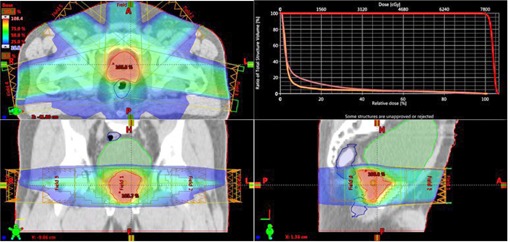
Isodose distributions in axial, coronal, and sagittal planes and DVHs of the involved structures for the reference prostate plan.

The above technique is applied to plan four clinical cases: three VMAT head and neck (HN) cases and a fixed gantry IMRT prostate case. The selected reference plan for a three‐arc HN study is displayed in Fig. 2. This is a clinically challenging case with 50 Gy prescribed to $V_{95}$ of the PTV. To meet the dosimetric constraints of the eyes, optic nerves, and chiasm, a sagittal arc is used along with two full coplanar arcs. The photon energy for the arcs is 6 M V. The other two HN cases have 70 Gy prescribed to $V_{95}$ of the PTV at 2.12 Gy/fraction. Two full coplanar arcs of 6 MV were used. For the prostate study, a typical five‐field IMRT plan with similar anatomy is used as the reference (Fig. 3). In the reference plan, 6 MV photon energy is used for all beams and the beam angles are 0°, 50°, 100°, 260°, and 310°, respectively. 78 Gy is prescribed to cover $V_{95}$ of the PTV in 39 fractions. For comparison, the resultant dose distributions of the autopiloting scheme are compared with the corresponding plans used for clinical treatments, which were obtained manually by a dosimetrist (signed off by a physician) and were deemed to be clinically optimal.

## III. RESULTS

### A. HN VMAT cases

**Figure 4:**
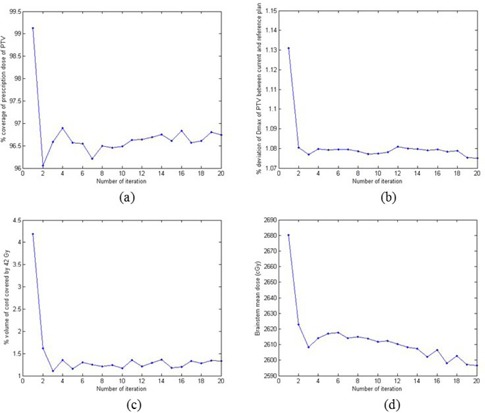
Change of a few dosimetric quantities as a function of outer‐loop iteration. Each iteration consists of tuning optimization parameters, decision making, optimization, dose calculation, and saving parameters to file. Panel (a) shows the percent coverage of prescription dose of PTV; Panel (b) shows the percent deviation of the maximum dose of PTV between the current and reference plans; Panel (c) shows the percent volume of the spinal cord covered by 42 Gy; Panel (d) shows the mean dose of the brainstem.

**Figure 5:**
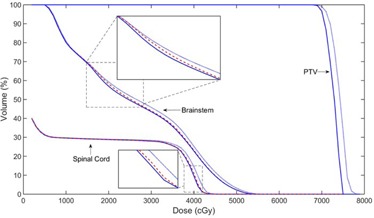
DVHs of a few structures at iteration #1 (dotted blue), #10 (dashed red), and #20 (solid blue). A systematic improvement in the DVHs of the brainstem and spinal cord is observed.

In Fig. 4, we show the progressive improvement of the PTV and OAR doses as a function of outer‐loop iteration for the first HN case. The improvement in doses of the involved structures is most prominent in the first few iterations. The dosimetric differences of the PTV and the spinal cord between the current and reference plans saturates after about eight iterations. However, the brainstem dose continues to improve until a later stage. The DVHs of a few relevant structures at different stages of iterative calculation are displayed in Fig. 5, which provides an overall picture of the iterative optimization process of the system.

**Figure 6:**
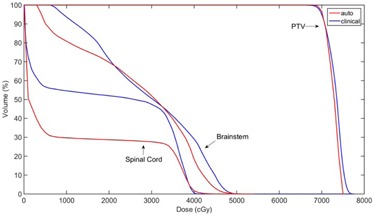
Final DVH of autopilot planning compared with clinical planning for the first HN VMAT case.

For comparison, the VMAT plan used for the patient's treatment is presented together with the current result. A comparison of DVHs between the clinical and autopiloted planning is shown in Fig. 6.

**Figure 7:**
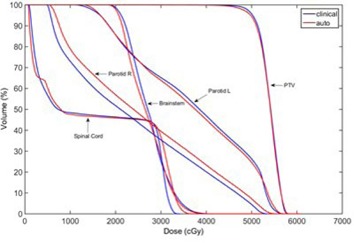
Final DVH of autopilot planning compared with clinical planning for the second HN VMAT case.

**Figure 8:**
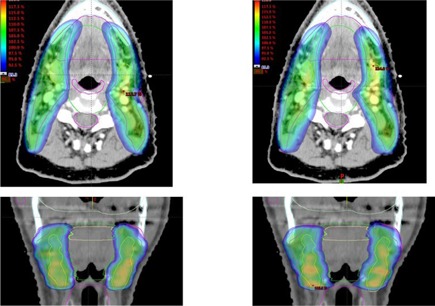
Side‐by‐side comparison of the isodose distributions of autopiloted (right) and clinical (left) plans for the second HN case.

**Figure 9:**
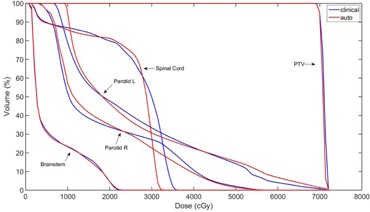
Final DVH of autopilot planning compared with clinical planning for the third HN case.

**Figure 10:**
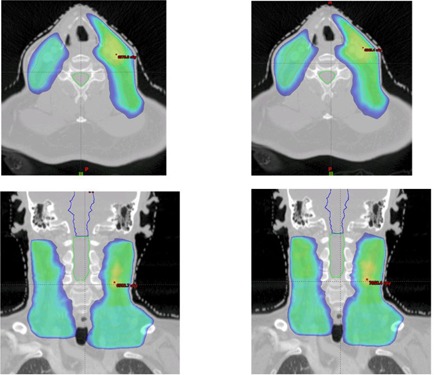
Side‐by‐side comparison of the isodose distributions of autopiloted (right) and clinical (left) plans for the third HN case.

Figure 7 shows the DVH comparison between the clinical and autopiloted planning for the second HN case. Figure 8 shows the isodose distributions of the two plans. Similarly, Figs. 9 and 10 show the DVH and isodose comparisons between the clinical and autopiloted planning for the third HN case.

It is interesting to see the minor discrepancy in the autopiloted and clinical plans. The former is obtained under the guidance of the reference solution, whereas the clinical plan was generated by a human planner independently. Clinically, it is known that interplanner variation occurs frequently in treatment planning, especially in some sophisticated cases like the one presented here. In reality, it is important to use high quality reference plan in autopiloted planning, even though post‐autopilot refinement of the treatment is feasible.

### B. Five‐field prostate IMRT

**Figure 11:**
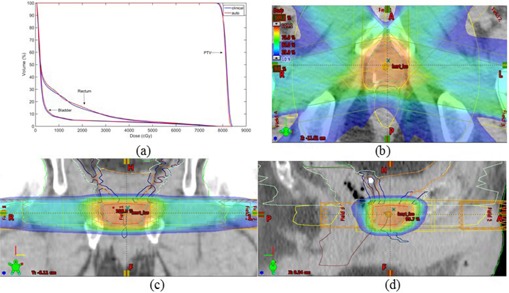
Panel (a) shos the final DVH of autopiloted planning as compared with manual planning for the prostate IMRT case. Panels (b)–(d): the isodose distributions of the autopiloted plan. The isodose distribution of the plan used for this patient's treatment is almost identical to the autopilot plan and is thus not shown here.

It takes ~20 iterations for the calculation to reach the optimum. The final solution is similar to the reference plan in terms of the overall isodose distribution. It is noted that, after the system reaches to its optimal solution, a small variation (1% to 3%) in one or more Eclipse parameters does not cause a noticeable change in the final dose distribution and DVHs, suggesting that the final solution is stable. The final optimal isodose distribution and DVHs for the case are shown in Fig. 11. Further improvement in the OARs could not be achieved without seriously sacrificing the target dose homogeneity. The bladder DVH of the resultant plan approaches to that of the reference plan easily because of more favorable anatomy. The rectum in the case under study is geometrically closer to the PTV as compared with that of the reference case, thus its dose shows a slightly larger variation from the reference plan.

### C. Computation time

Computationally, it takes about 3 hrs to complete a plan selection process, but this can be improved with more efficient programming, and, in the future, better integration with the Eclipse. We find that about 70% of the time is spent by the Eclipse on tasks such as optimization and dose calculation, 15% for reading from files and computation, 10% for inputting new values, and 5% for buffering among actions. An algorithm that iteratively modifies the beams and objective function parameters altogether should, estimated based on the number of outer‐loop/inner‐loop calculation, be 15 times more efficient, but this would entail tackling/modifying the objective function of the commercial TPS and becomes practically impossible. Nevertheless, comparing with the current manual trial‐and‐error planning process, which could take days of a dosimetrist's time for clinically challenging cases, the proposed method eliminates the need for modifying the optimization parameters and other time‐consuming tasks, such as adding Boolean structures and converting isodose curves into structure necessary to shape dose distribution toward meeting the clinical expectation. The entire calculation is done without any user invention. It thus represents a step forward in inverse planning technique.

## IV. DISCUSSION

In current practice, treatment planning involves testing a large number of physically realizable solutions. Intertwined interactions of TPS optimization and planner's adjustment of TPS planning parameters are needed to obtain a sensible plan. While it is desirable to automate the planning process through development of more intelligent optimization algorithm,^(3–5,8,9,28,29)^ most TPS systems are far from being ideal and multiple trial and errors are necessary to obtain a clinically acceptable plan.^3^, ^4^, ^29^ How to make TPS better comprehend the planner's goal has been an active research topic since the early days of inverse planning research^(3,4,6,25,30–36)^ and its perspective remains elusive. Instead of attempting to improve the optimization algorithm of the TPS, which is clearly out of the control of a user, the proposed platform and the use of an out‐of‐the‐TPS decision function here allows us to imitate a planner's interactive planning process to search for the optimal solution that would otherwise require much more manual effort. The approach is quite general and should be applicable to facilitate the treatment planning of other modalities such as brachytherapy,^(37)^ proton therapy,^38^, ^39^ station parameter optimized radiation therapy (SPORT),^29^, ^40^ gamma knife,^(41)^ breast planning^(42)^ or similar.

As described in the Material and Methods section C, autopilot planning entails the use of a reference plan to drive the optimization process toward a clinically sensible solution. How to better define a set of clinically relevant geometric criteria for finding the best possible reference case(s) is an on‐going research issue in knowledge‐based planning. This can be realized in our study by selecting a reference plan(s) that leads to positively signed difference for points entering the OARs (meaning that the “distance of the OAR to the PTV” in the current plan is larger than that of the reference plan). Based on our clinical experience, being able to pilot the planning process to a point that is sufficiently close to be clinically reasonable/acceptable takes up the most portion of the planning efforts and thus represents the single most challenging aspect of clinical inverse treatment planning process. Even if a reference plan is not the optimal solution, it is useful to guide us to the proximity of optimal plan. In practice, refining a plan that is “almost there” requires much less effort as compared to planning from “scratch”. In our implementation, we have made effort to make the autopiloted planning to generate a plan that may exceed the quality of the selected reference plan.

We note that knowledge‐based treatment planning has been one of the subjects actively studied recently.^(5,43–45)^ The approach can be roughly classified into three categories: 1) those that predict the weighting factors for different structures using machine learning algorithms; 2) those that generate initial solutions for the “warm start” of the subsequent optimization; and 3) those that estimate the desired DVH curves to be achieved by the subsequent optimization. Practically, none of the three categories provides a turn‐key solution to automate the treatment planning, as the beam parameters that produce the best possible dose distribution still need to be optimized for patient treatment. In the future, it seems to be useful to combine the autopilot planning framework proposed here and the knowledge‐based estimation of reference plan(s) to make the autopiloted planning more computationally efficient.

Generally, Coded UI does not rely on the absolute coordinates of the DVH data or GUI control buttons on the screen to perform the recording/playback, which is different from an alternative approach recently proposed by Tol et al.^(36)^ Instead, it relies on the relative positions of the buttons. Therefore, controls can change their physical locations (layout) as long as their relative locations are maintained. If the GUI is modified in a version change of TPS to the point where certain controls are interchanged, or deleted or new controls are added, then even a manual planner would need to be trained again. In this scenario, we would need to rerecord some of the actions. Recording a new action can easily be added to the preexisting library, since the code is automatically generated in Coded UI. Consistent naming of structures with approach consistent with the emerging ontology convention is important. We note that, while Coded UI is designed for the Windows environment, the principles and strategies proposed here are quite broad. The research experience gained in this platform can be translated to other platforms by other researchers to improve the manual trial‐and‐error process of inverse planning.

Other operating systems, such as Unix and Linux, have similar record‐and‐playback services (UNIX Session Recorder, Sikuli, Linux Desktop Testing Project).^(46)^ In practice, Windows platform is employed by a large number of TPS and other clinical application software. Thus, the presented strategy can be applied directly to facilitate clinical tasks or workflow. Finally, in our approach the C# program acts like a human planner, iteratively interacting with the TPS. Thus, similar to the fact that a computer used by a manual planner is not expected to concurrently run other tasks, the TPS computer cannot be used for other purposes while the C# program is running.

In our implementation, the data (such as the DVHs data) exchange between the inner‐ and outer‐loop optimizers are realized mainly through export/import. In addition to these options, assertion statements can be used to extract the state of the Eclipse system. The information updates us in real time about which operations are complete, which still on‐going, and which are running into an error. It also helps to capture the cause and effect of different actions before moving the calculation to the next step.

The reported method may have useful implications to medical physics research and clinical practice. Currently, the development of treatment planning algorithm(s) in research community is often done in simplified software platforms^47^, ^48^ without the knowledge of some important geometric and physical factors,^47^, ^49^ which makes it difficult to experimentally validate the results using the clinical linacs, to compare the results with current practice, and to translate the research into clinical practice. The gap between research and clinically used TPS has increased dramatically over the years and TPS becomes essentially a black box to the researchers. The approach here enables researchers to leverage the sophisticated software subroutines existing already in a commercial TPS. The described technique enables us to utilize various software subroutines/functions in a clinical grade TPS without going into the details of their implementations, which may take years of professional engineers’ efforts to develop and validate. With the proposed approach, researchers can focus their efforts in testing their new ideas instead of spending a huge amount of efforts to “redevelop” the software subroutines/functions that already exist in a commercial TPS. By avoiding “reinventing the wheels” or repeating some well‐known tasks, such as dose calculation and image registration, the researchers can focus their efforts on high‐level research issues. The programming environment described here is highly interactive, which makes it easy when it comes to principle testing and prototyping. Another important advantage of the technique here is that it may facilitate the translation of research to clinical practice because of the elimination of intermediate layers/issues as mentioned above. In terms of applications, the implemented two‐loop optimization represents only one of many possible applications of the proposed strategy. Coded UI is designed to interact with the user interface in the Windows environment, thus the approach is applicable to streamline other clinical tasks that require a series of operator software interactions in TPS.

## V. CONCLUSIONS

We have demonstrated the use of recorded user software interactions for a commercial TPS in autopilot of VMAT/IMRT treatment planning. The approach makes it easy to utilize the software tools of a clinical TPS for development of new applications and presents an uncharted area for research and applications. The strategy allows programmatically controlled execution of tasks that require a series of commands and should thus improve the clinical workflow. An autopilot optimization algorithm with incorporation of prior knowledge is implemented in combination with Eclipse TPS. The algorithm presents a practical way to mimic the decision‐making process of a planner and to pilot the plan optimization toward the reference plan, thus reducing the need for trial and error in treatment planning. The autopilot method promises to simplify the clinical treatment planning. Finally, the approach should be extendable to the automation of other tasks in using a software tool.

## ACKNOWLEDGMENTS

We wish to thank Drs. Yong Yang, Masoud Zarepisheh, Karl Bush, Barris Ungun, Stephen Boyd, and Yinyu Ye for many useful discussions. This work is partially supported by NIH (5R01 CA176553 and 1R01 EB016777) and Varian Medical Systems.

## COPYRIGHT

This work is licensed under a Creative Commons Attribution 3.0 Unported License.
